# Plagiarism Reimagined

**DOI:** 10.1093/function/zqad014

**Published:** 2023-03-23

**Authors:** R A North

**Affiliations:** Faculty of Biology, Medicine and Health, University of Manchester, Oxford Road, Manchester M13 9PT, UK

Once upon a time, scientists wrote with quills, then pens, then typewriters. There was often someone else well trained in the art, often poorly paid, who sat behind the typewriter, working from a yellow paper legal pad, or even from a simple tape recorder. That was the time when one went to the library in person, when copying machines were not available or very expensive, and when one made notes about another paper on an index card, which fitted into a metal box, where it sometimes remained for a long time without being viewed again. It was in that period when I received, in the mail, a request to review a manuscript from the journal *Life Sciences*.

The year before, Marcello Tonini and I had published a paper in the *British Journal of Pharmacology*,^1^ which described the hyperpolarizing action of morphine on neurons of the myenteric plexus. This is Auerbach’s plexus, one of the two major ganglionated nerve plexuses in the wall of the intestine: A few years before, working with Syogoro Nishi in Chicago, I had reported the first intracellular recordings from these neurons.^2^

A short segment of guinea pig ileum had been widely used over the years as an in vitro bioassay for morphine and related compounds: Electrical stimulation of the myenteric neurons caused the release of acetylcholine, which contracted the longitudinal muscle, and this contraction could be readily recorded on a kymograph (smoked drum) or, later, a pen recorder. By studying a wide variety of morphine-like compounds, agonists and antagonists, it had been concluded that the receptor on the myenteric neurons was extremely similar to the receptor through which morphine evinced its analgesic and other effects in man.^3^ The bioassay had been a key component of the recent identification of the first endogenous opioids, the pentapeptides met- and leu-enkephalin.^4^

The manuscript that came for my review from *Life Sciences* described the hyperpolarizing action of enkephalins of myenteric neurons, studied by intracellular microelectrode recording. It began:

The isolated guinea-pig ileum preparation with myenteric plexus are now widely used as one of the model systems in the investigation of narcotic analgesics. It has been reported that both the acetylcholine release from this preparation and contractile response of the longitudinal muscle layer to electrical stimulation of its nerves are greatly depressed by narcotic analgesics. These actions of narcotic analgesics correlate well. . . stereospecific.

I was strongly reminded of my paper with Tonini,^1^ which began:

The guinea-pig isolated ileum and the myenteric plexus-longitudinal muscle preparation are now widely used as model systems in the investigation of narcotic analgesics. Both the acetylcholine output from these preparations and the contractile response of the longitudinal muscle layer to electrical stimulation of its nerves are greatly depressed by narcotic analgesics. These actions of narcotics correlate well. . . stereospecific.

That was just the beginning of the Introduction, but many further sections throughout the manuscript were also copied verbatim from our paper. On the other hand, the actual findings, and indeed some of the Figures, seemed original: They showed that enkephalins had pretty much the same hyperpolarizing action on myenteric neurons that we had previously reported for morphine and its analogs. Not quite sure whether to be flattered or concerned, I wrote to Tom Burks, the Editor of *Life Sciences*, and pointed out the substantial transplantation of text from our paper to this submission to *Life Sciences*. He telephoned me to explain that this was utterly unacceptable, and that the authors had been informed never to submit any further work to his journal.

Naturally, I continued to follow the associated literature, through my weekly visits to the library, and later that year, I noticed a paper published in *Neuroscience Letters*.^5^ It began:

The isolated guinea-pig ileum preparation with myenteric plexus are widely used as one of the model systems in the investigation of narcotic analgesics [1]. It has been reported that both the acetylcholine release from this preparation and the contractile response of the longitudinal muscle layer to electrical stimulation of its nerves are greatly depressed by narcotic analgesics [2–4]. These actions of narcotics that correlate well. . . stereospecific [8].

Reading on, I found many further sentences in the *Neuroscience Letters* article^5^ that were lifted intact from our earlier paper in the *British Journal of Pharmacology*,^1^ with the word enkephalin substituted for morphine: Almost one-half of the *Neuroscience Letters* paper had been copied from our previous work. I wrote to Manfred Zimmermann, the Chief Editor of *Neuroscience Letters*. He seemed concerned by the situation and eventually published a “Disclaimer.”^6^ After referring to the identity of statements in the two papers,^1,5^ it continued:

While the Editors and Publishers of Neuroscience Letters take every precaution to ensure that material submitted to them has not been published in a previous form elsewhere, it is virtually impossible for us to ensure that such problems do not arise. The Editorial Board and Publishers, therefore, feel compelled to refer readers of the article in this Journal to the second article which was obviously published some time in advance.

Several years later, I was telephoned quite out of the blue by a Dean in a North American university, who explained that he was handling an investigation into one of his academic staff. He simply asked whether I could confirm this earlier case of plagiarism.

How fortuitous is it to receive for review a paper that copies one’s own earlier work, and in a day when such plagiarism required hard work, careful copying, and retyping. And how surprising to see the same piece work eventually appear elsewhere. In today’s world, it is enough to request an AI-based text generator such as ChatGPT to provide the required manuscript.^7,8^ Sad to say, the motivation behind such dishonesty is little altered by the recent facilitation of the technology. Rather, that motivation has been greatly strengthened by the inexorable application of simplistic numerical methods to assess the quality and/or impact of an individual’s scientific work, such as the *h*-index. And going to the library was very rewarding: Often enough, it was an unexpected paper quite unrelated to your field that provided the greatest intellectual stimulus.
